# Virtual reality interventions for victims of crime: A systematic review

**DOI:** 10.1002/jts.22810

**Published:** 2022-02-28

**Authors:** Giovanna Parmigiani, Leda Tortora, Gerben Meynen, Gabriele Mandarelli, Stefano Ferracuti

**Affiliations:** ^1^ Department of Human Neurosciences “Sapienza” University of Rome Rome Italy; ^2^ School of Nursing and Midwifery Trinity College Dublin Dublin Ireland; ^3^ Willem Pompe Institute for Criminal Law and Criminology Utrecht University Utrecht The Netherlands; ^4^ Faculty of Humanities Vrije Universiteit Amsterdam Amsterdam The Netherlands; ^5^ Section of Criminology and Forensic Psychiatry Department of Interdisciplinary Medicine University of Bari Bari Italy

## Abstract

In the forensic field, most studies employing virtual reality (VR) interventions have focused on offenders. The validity and safety of VR applications for victims of crime are still unclear. Following PRISMA guidelines, a systematic review on VR interventions for crime victims was performed to assess the efficacy, acceptability by patients, and cost‐effectiveness of these interventions compared to in‐person care. We identified 34 potentially eligible studies from 188 records obtained from database searches (Medline/Pubmed, CINAHL, PsycINFO, Web of Science, and Scopus); four additional articles were identified via alternative sources. In total, nine articles were included for the qualitative synthesis. Patient satisfaction with VR interventions was found to be equivalent to face‐to‐face interventions. Both VR exposure and control groups found relief from posttraumatic symptoms, with differences either statistically insignificant or in favor of VR. Despite the increased costs linked to the technology required, VR appears to be a promising alternative to in vivo exposure, but further research is needed. Limitations of the review include the varied experimental protocols, which did not allow us to conduct a quantitative analysis and comparison of findings across different studies, and the generally poor quality of the studies included. Further research, preferably in larger groups, is needed to shed more light on the effectiveness of VR interventions for traumatized victims of crime.

The term *virtual reality* (VR) refers to a technology aimed at simulating real‐life situations in a tridimensional computer‐generated environment with which users can interact as if they were in the real world. Ideally, VR users should not be able to distinguish the sensory experiences, feelings, and interactions associated with the virtual world from those elicited by the real world (Parsons et al., 2017). The feeling of being in the virtual environment, even if one is physically in another reality, is called *presence* (Schuemie et al., 2001). Similarly, the subjective experience that a virtual character exists in the environment is called *social presence* (Parsons et al., 2017). Finally, the VR user's experience of being wholly absorbed by the simulated environment, momentarily “forgetting” their embodied presence in the physical world, is referred to as *immersion* (Kellmeyer et al., 2019). Because of presence and immersion, the VR environment ideally triggers the same behavior as its real equivalent (Alsina‐Jurnet et al., 2011). VR technologies have also been applied in health care, particularly mental health care. VR makes it possible to expose patients to stimuli and places that would otherwise be difficult to access and offer a more confidential setting than in vivo exposure (Botella et al., 2015). Among the advantages of VR over in vivo exposure therapy, VR allows for the replication of different physical or situational environments to treat various mental disorders (Botella et al., 2015). Importantly, the VR environment can be adapted to a patient's needs to help them modify behaviors, thoughts, and emotions (Botella et al., 2015). Relatedly, therapists can control the virtual situation to a high degree; for example, they can prevent the occurrence of unpredictable events or repeat a specific exposure task as many times as necessary. However, the realistic features of VR treatment also entail risks. For instance, when treating posttraumatic stress disorder (PTSD), previous research has suggested that VR exposure confers a risk of retraumatization instead of the successful processing of a traumatic experience (Eichenberg, 2010). Still, VR therapy is scarce: Therapists tend to have limited access to suitable VR systems, and there is also a lack of training that currently prevents wider use in clinical settings (Botella et al., 2015).

In the mental health field, VR interventions have shown positive effects in patients affected by specific phobias (Botella et al., 2017), PTSD (Botella et al., 2015), psychoses (Craig et al., 2018; Veling et al., 2014), and eating disorders (Ferrer‐Garcia & Gutierrez‐Maldonado, 2012), as well as offenders in forensic mental health facilities (Kip et al., 2018). VR has also been applied for individuals who have been the victim of or witness to a crime, such as military sexual trauma (Loucks et al., 2019), who often report PTSD and anxiety‐related disorders. Regarding the treatment of PTSD, two main types of VR environments have been identified: a VR environment with very specific and realistic situations (Difede et al., 2007) and a flexible VR environment that uses symbolism to represent traumatic events (Baños et al., 2011). A clear advantage of specific and realistic VR environments is their hyperrealism, as the traumatic situation is represented with very specific details; however, employing these treatments in daily clinical practice is costly, as different virtual environments are required to treat diverse problems (e.g., terrorist attacks, sexual assault; Baños et al., 2011; Botella et al., 2015). The flexible type of VR environment is an adaptable system wherein traumatic events are symbolically represented using different tools, such as symbols, pictures, music, sounds, and video. The main advantage of this type of VR environment is its flexibility in representing any traumatic event, whereas its main weakness is that for some PTSD populations, a more specific and realistic VR environment might be more suitable (Botella et al., 2015).

In the forensic field, most studies employing VR interventions have focused on offenders, from predicting reoffending risk (Fromberger, Jordan, et al., 2018; Fromberger, Meyer, et al., 2018; Klein Tuente et al., 2020; Nee et al., 2019; Nee et al., 2014), to offending rehabilitation and integration (Fromberger, Jordan, et al., 2018; Kip et al., 2018; Kip, Kelders, Bouman, et al., 2019; Kip, Kelders, Weerink, et al., 2019; Renaud et al., 2010; van Rijn et al., 2015). Only a few studies have focused on the victims of crime, and these examinations have led to contrasting results (Botella et al., 2010; Cárdenas‐López et al., 2014, 2013, 2015; Difede et al., 2007, 2014; Jouriles et al., 2014; Loranger & Bouchard, 2017; Peskin et al., 2019). The goal of the present paper is to provide a systematic review on VR interventions for crime victims and assess the validity, patient acceptability, cost‐effectiveness of this treatment modality compared to in‐person care, with the aim of helping providers to be better equipped to understand advantages and potential drawbacks of VR interventions.

## METHOD

This systematic review was conducted following the Preferred Reporting Items for Systematic Review and Meta‐Analyses (PRISMA) guidelines (Liberati et al., 2009).

### Literature search

We used a systematic search strategy to identify relevant articles. We conducted a two‐step literature search on April 23, 2020. As a first step, the Medline/Pubmed, CINAHL, PsycINFO, Web of Science, and Scopus databases were searched, with the following search terms: (victim* OR abused) AND (“crime*” OR “assault” OR “violen*” OR “interpersonal violen*” OR “rape” OR “stalking” OR “harassment” OR cybercrime) AND (“virtual reality” OR “augmented reality” OR “artificial reality” OR VR). The second step involved two authors performing an additional electronic search based on the manual mining of the reference lists of the retrieved articles. We then screened the abstracts of the articles identified during these two steps for eligibility, and articles deemed eligible for inclusion in the review were then further assessed based on a full‐text reading. Discrepancies were resolved through consensus with a third author.

### Inclusion and exclusion criteria

We examined the following outcomes: acceptability and patient satisfaction, efficacy, and cost‐effectiveness. The inclusion criteria were (a) randomized controlled trials (RCTs), nonrandomized control trials (NCTs), or pre–post studies; (b) the investigation of the effects of VR interventions for victims of crime; and (c) adult samples (i.e., participants were 18 years of age or older). We excluded articles written in languages other than English, Italian, or Spanish; reviews, retrospective studies, and case reports; and articles for which we were unable to obtain the full text even after contacting the corresponding author.

### Data extraction

Titles and abstracts were screened by two reviewers independently, in duplicate, to determine whether the retrieved studies met the previously outlined inclusion criteria. Full texts were obtained for studies that appeared to meet the inclusion criteria and those for which a decision could not be made from the title and/or abstract alone, and a detailed review of the criteria was performed. Full texts were independently assessed for eligibility by two reviewers, and discrepancies were resolved by an initial discussion or consultation with a third reviewer, when required, until a complete consensus was reached.

A standardized form was used to extract data from the included studies to assist in the evaluation of study quality and synthesis of evidence. Extracted information included study focus, participant characteristics, details of the intervention and control conditions, study methodology, dropout rate, outcomes and assessment times, and information needed for risk of bias (RoB) assessments. Extraction was completed by two reviewers independently, in duplicate. A third reviewer was consulted when needed.

### Quality evaluation

The quality of the included studies was assessed using the RoB assessment tool developed by the Cochrane Collaboration for randomized studies (Higgins & Green, 2011) and the Risk of Bias in Nonrandomized Studies of Interventions (ROBINS‐I) tool for nonrandomized studies (Sterne et al. 2016). The RoB assessment identifies possible sources of bias in randomized control trials, such as selection bias (i.e., random sequence generation, allocation concealment), performance bias (i.e., blinding of participants and personnel), detection bias (i.e., blinding of outcome assessment), attrition bias (i.e., incomplete outcome data), reporting bias (i.e., selective reporting), and other bias (i.e., bias due to problems not covered by the other points). The ROBINS‐I is used to investigate seven domains through which bias might be introduced into a nonrandomized trial, categorized as occurring preintervention (bias due to confounding and bias in the selection of participants into the study), at intervention (bias in classification of interventions), and postintervention (bias due to deviations from intended interventions, bias due to missing data, bias in the measurement of outcomes and bias in the selection of the reported result).

## RESULTS

**FIGURE 1:**
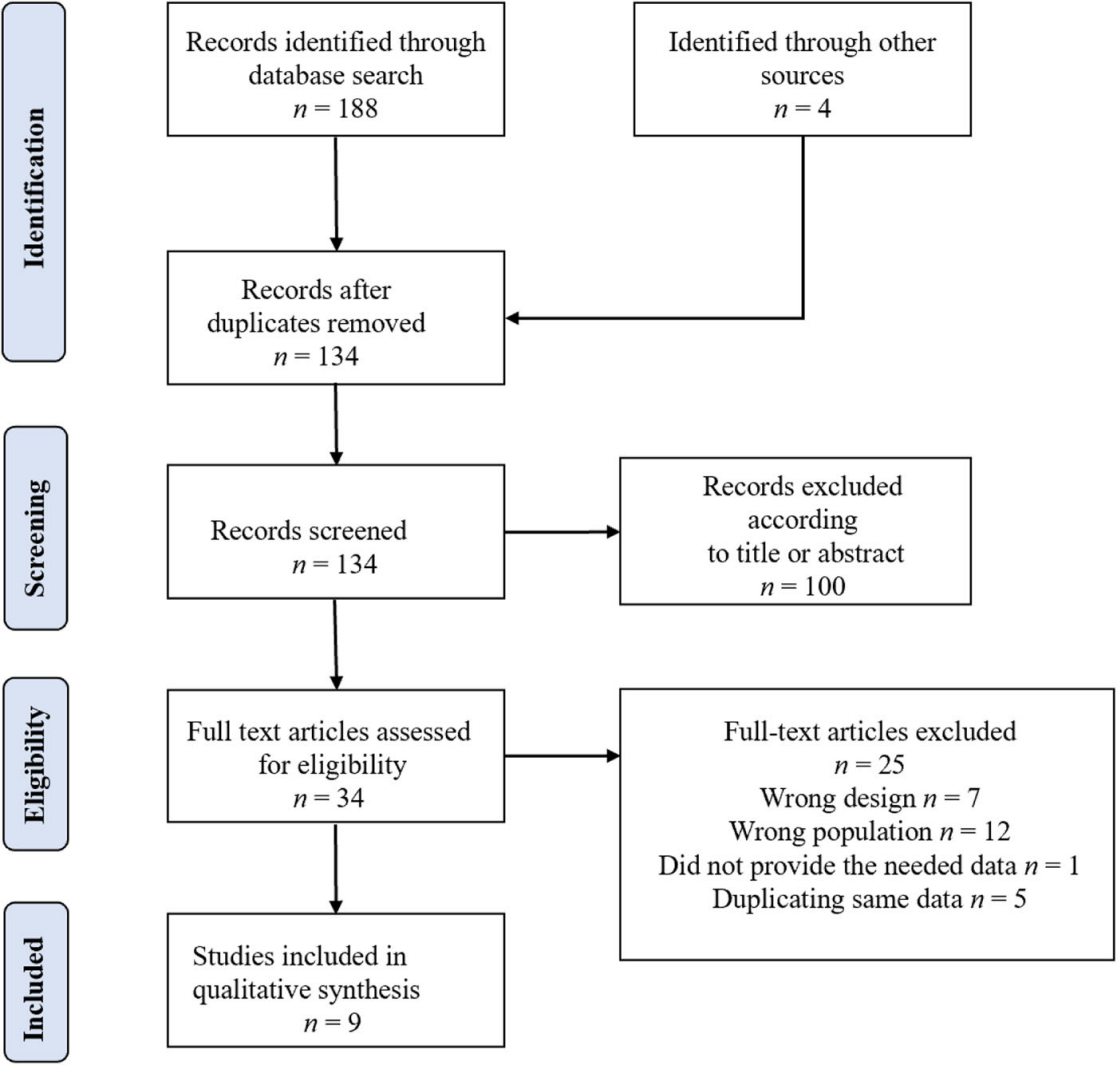
PRISMA flow diagram

We identified 34 potentially eligible studies from 188 records obtained from the selected databases and four obtained from alternative sources. After reviewing the full content of the papers, 25 articles were excluded for several reasons: seven were case descriptions, conference proceedings, or reviews; 12 did not examine a population of interest (i.e., adult victims of crime); one did not provide the necessary data, which were unable to obtain after contacting the study author; and five contained duplicate data. The process of identifying eligible studies is outlined in Figure 1. For the list of the excluded studies see the Supplementary Materials.

### Studies, participants, and treatment characteristics

The characteristics of the included studies are summarized in the Supplementary Materials. Four studies (Botella et al., 2010; Cárdenas‐López et al., 2013; Loranger & Bouchard, 2017; Peskin et al., 2019) were RCTs, 1 was a NCT with a quasi‐experimental design (Difede et al., 2007), and four were pre–post studies (Cárdenas‐López et al., 2015, 2014; Freeman et al., 2014; Jouriles et al., 2014).

Eight studies (Cárdenas‐López et al., 2014, 2013, 2015; Difede et al., 2007; Freeman et al., 2014; Jouriles et al., 2014; Loranger & Bouchard, 2017; Peskin et al., 2019) used a specific and realistic virtual environment, whereas one study (Botella et al., 2010) used a flexible and adaptable VR environment that employed symbolism to represent the traumatic event (i.e., EMMA's world). Three studies were conducted in the United States (Difede et al., 2007; Jouriles et al., 2014; Peskin et al., 2019), one in Canada (Loranger & Bouchard, 2017), one in Spain (Botella et al., 2010), one in the United Kingdom (Freeman et al., 2014), and three in Mexico (Cárdenas‐López et al., 2014, 2013, 2015).

Regarding the assumption of pharmacological therapy, only four studies reported on this variable. Half of one study sample was taking medication (Botella et al., 2010); three out of 13 patients in another study were taking selective serotonin reuptake inhibitors (SSRIs; Difede et al., 2007); one study included no patients who were taking medication (Cárdenas‐López et al., 2014); and 56% of participants in the study by Peskin et al. (2019), as reported by Difede et al. (Difede et al., 2014), were on psychotropic medications.

### Quality evaluation

**FIGURE 2:**
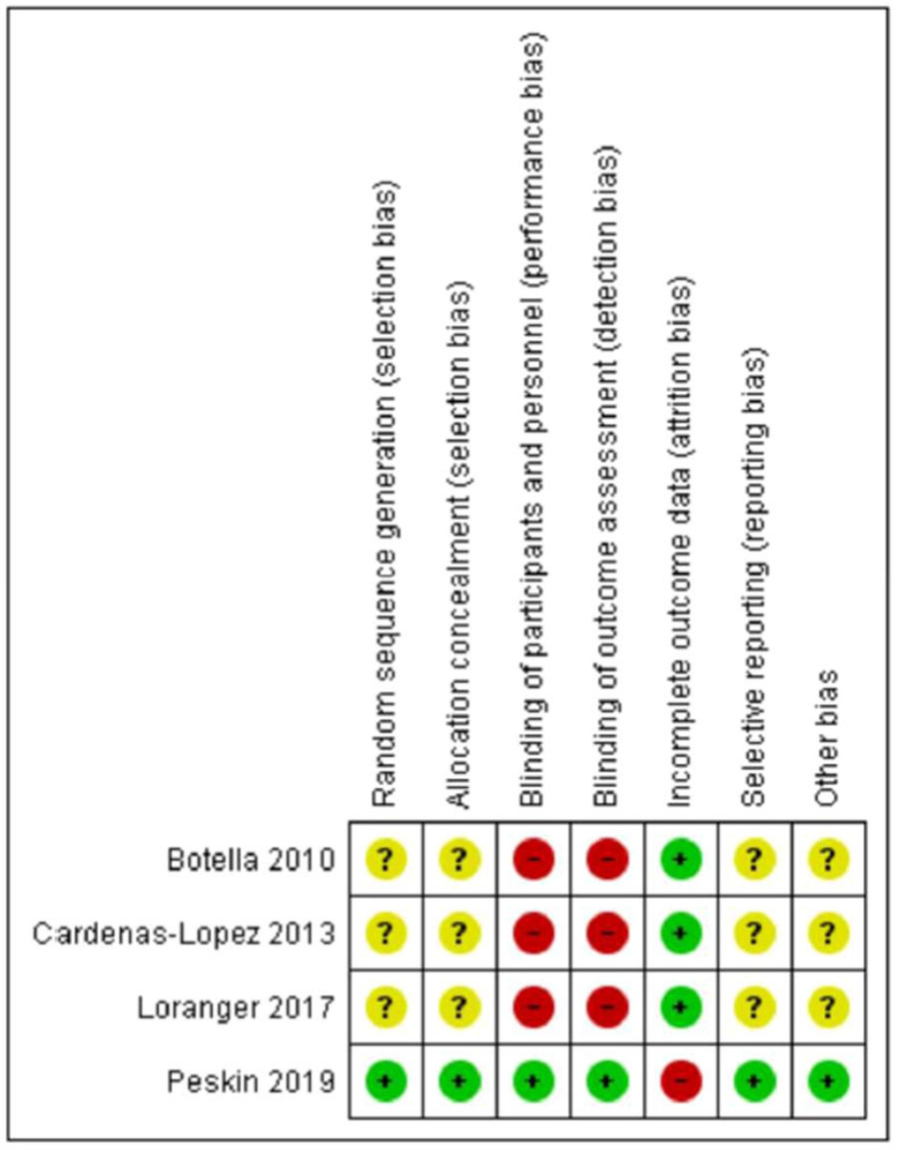
Risk of bias summary. Note. “+” indicates a low risk of bias. “–” indicates a high risk of bias. “?” indicates an unclear risk of bias

Figure 2 summarizes the different aspects concerning the methodological quality of the RCTs. Among the included RCTs, the overall quality was poor. Only one study (Peskin et al., 2019) reported the method used for generating the random sequence, as outlined in the published protocol, and noted whether the method used to hide the sorting of patients allowed for the prediction of patient distribution into groups in the study by Difede et al. (2014), which described the methodology. Two of the studies did not use a blind evaluator (Botella et al., 2010; Loranger & Bouchard, 2017), one study made use of a blind evaluator (Peskin et al., 2019), and one did not provide information about whether the evaluator was blinded (Cárdenas‐López et al., 2013). In one study, the differential attrition was judged as problematic (i.e., 25% of patients dropped out in the placebo group vs. none in the intervention group). The study protocol was available only for one study (Peskin et al., 2019).

Table 1 shows ROBINS‐I RoB of nonrandomized studies. One study (Difede et al., 2007) was evaluated to have an overall critical risk of bias, three studies (Cárdenas‐López et al., 2014; 2015; Jouriles et al., 2014) had a serious overall risk of bias, and one study (Freeman et al., 2014) had a moderate overall risk of bias.

**TABLE 1:** Risk of bias judgments in nonrandomized studies of interventions (ROBINS‐I) assessment

Study	Confounding	Selection of participants	Classification of interventions	Deviation from intended interventions	Missing data	Measurement of outcomes	Selection of reported results	Overall^a^
Difede et al., 2007	Critical	Low	Critical	Critical	Moderate	Moderate	Low	Critical
Cárdenas‐López et al., 2014	Serious	Low	Moderate	Low	Low	Moderate	Low	Serious
Cárdenas‐López et al., 2015	Serious	Low	Low	Low	Low	Moderate	Low	Serious
Freeman et al., 2014	Moderate	Low	Low	Low	Low	Low	Low	Moderate
Jouriles et al., 2014	Serious	Low	Serious	Low	Low	Moderate	Low	Serious

*Note*: “Low” indicates that the risk of bias is comparable to that in a well‐performed randomized trial. “Moderate” indicates that the risk of bias is sound for a nonrandomized study but not comparable to a rigorous randomized trial. “Serious” indicates the presence of important problems. “Critical” indicates that the risk of bias is too problematic to provide any useful evidence on the effects of the intervention.

^a^ Overall risk of bias rating is equal to the most severe level of bias found in any domain.

### Acceptability and patient satisfaction

Few studies reported on patient satisfaction and acceptability. One study that examined patient satisfaction demonstrated no difference between VR interventions and treatment as usual (Cárdenas‐López et al., 2013). Another study, which did not use a control group, reported that all patients who received VR interventions were satisfied with the received treatment (Cárdenas‐López et al., 2014).

### Efficacy

Among the studies that examined treatment efficacy, VR interventions showed a higher efficacy compared to imaginary exposure therapy (IET; Cárdenas‐López et al., 2013) and waitlist conditions (Difede et al., 2007); however, Botella et al. (2010) found no differences between cognitive behavioral therapy (CBT) and VR exposure. Freeman et al. (2014) found that responses to VR predicted the severity of paranoia and PTSD symptoms as assessed using standard measures 6 months later. Finally, two studies by Cardenas and colleagues (Cárdenas‐López et al., 2015, 2014) reported that VR treatment was effective in treating PTSD patients, with all patients showing a clinically significant improvement in PTSD symptoms (i.e., a score improvement greater than 30% on a measure of PTSD symptoms) after 12 weeks of treatment.

### Cost‐effectiveness

Few studies mentioned the potential cost implications of VR interventions. In three separate studies, Cárdenas‐López et al. (2014, 2013, 2015) briefly discussed the idea that virtual reality interventions could be more cost‐effective than other modalities, but they did not include this variable or outcome in their analyses. Thus, we could not find empirical support for this statement.

## DISCUSSION

This systematic review of nine studies on VR interventions for victims of crimes showed a general trend toward benefits conferred from immersive VR in reducing PTSD symptoms when compared to control conditions (Cárdenas‐López et al., 2013; Difede et al., 2007) and in pre–post intervention studies (Cárdenas‐López et al., 2015, 2014). In addition, immersive VR appears to be predictive of paranoia‐related and PTSD symptom severity in crime victims 6 months following treatment (Freeman et al., 2014) and can be useful to create a safe and realistic environment for gradual exposure to a sexual assault scenario (Loranger & Bouchard, 2017) as well as for role plays designed to help college women resist sexual attacks (Jouriles et al., 2014). Our findings are consistent with reviews of VR use in patients affected by PTSD (Botella et al., 2015; Goncalves et al., 2012; Motraghi et al., 2014) even though there is no available literature specifically focused on VR interventions for victims of crime. Still, the extent to which VR could be more effective than traditional treatments remains unclear, as does whether this modality is preferable for certain subgroups of victims.

Regarding victims of crime, the possibility of retraumatization is a marked concern. More research is needed to better understand the size of this risk and how it can be diminished or avoided. Still, in the absence of such research, it is plausible that symbolic versus realistic VR interventions could be safer in this respect, as they differ in important ways from the actual traumatic event and have demonstrated similar results. Another option might be to use a realistic version of the event but with some modifications that make it less similar to the traumatic event and can be tailored to the individual victim's preferences (e.g., the appearance of avatars). This may also signal the importance of involving actual victims in the development of VR interventions designed to help this population with regard to the process and potential functionalities. In general, from an ethical perspective, researchers have argued that with respect to VR in medicine, vulnerable patient voices should be included in the design phase (Kellmeyer et al., 2019).

In the included studies, satisfaction, when measured, was either equivalent to face‐to‐face therapy (Cárdenas‐López et al., 2013) or at least reported by patients undergoing VR interventions (Cárdenas‐López et al., 2014). This suggests that, overall, victims of crime find mental health care delivered via VR to be at least as acceptable as traditional, in‐person treatment. However, to date, few universities or academic centers offer any specific training in the field of cyberpsychology or “e‐health” for mental health providers, and, consequently, such interventions are scarce in the mental health field.

VR interventions are relatively costly because of the expensive equipment needed to administer them, such as a powerful computer with a good graphics card and head‐mounted display (Cornet & Van Gelder, 2020). Still, only three studies mentioned cost‐effectiveness (Cárdenas‐López et al., 2014, 2013, 2015), and the authors of these studies pointed to the cost benefits of VR procedures, likely due to the potentially high costs and low feasibility related to in vivo exposure. However, the authors failed to provide data supporting this statement; thus, further research is needed. Regarding anxiety treatments, Fodor et al. (2018) emphasized that, even though it seems “intuitive” that VR treatment would be cost‐effective compared to typical treatments, research has not yet provided evidence for this statement. The authors point to the fact that the cost‐effectiveness might depend on the disorder being treated. For a patient with a fear of flying, they argue, VR treatment may be much cheaper than a plane ticket, but for fear of heights, this might not be the case, as relevant exposures might be more accessible.

In addition, the cost of technology overall continues to decrease due to innovations in the market, and this will likely result in VR interventions becoming even more cost‐effective in the future. It is important to note that unlike some other health care technologies, such as magnetic resonance imaging and computed tomography scans, VR applications are used and developed predominantly outside of medicine. Some observers have predicted that health care applications will only comprise approximately 15% of the total VR and augmented reality market by 2025, whereas the consumer market will comprise 50% (Keswani et al., 2020). This means that the advancement of VR may be driven not only by research and development in health care but also by developments in the consumer market. This could also reduce the costs of VR in mental health care.

Finally, an important advantage of VR is that it can bring people together across distances. The patient and therapist do not necessarily have to be in one room, one building, one city, or even one country. This confers several benefits. Given the current COVID‐19 pandemic, it is necessary for individuals to be able to receive treatment at home from a distant therapist. But the technology also opens the possibility of specialized VR centers that treat victims in a larger region. This could be helpful to deal with the current—possibly temporary—scarcity of trained therapists.

There are several limitations of the present systematic review that make the current findings preliminary rather than conclusive. Because of the varied experimental protocols, it was not possible to conduct quantitative analyses and comparisons of the findings across different studies. In addition, several methodological issues limited the strength of the current conclusions: Most of the RCTs omitted data concerning random sequence generation, allocation concealment, blinding procedure, and description of incomplete outcome data, and only one included article published the study protocol (Peskin et al., 2019). Regarding the nonrandomized trials, the overall risk of bias ranged from critical to moderate. Moreover, the sample sizes were relatively small, with a maximum of 30 participants for RCTs (Loranger et al., 2017) and 106 for pre–post studies (Freeman et al., 2014). There is also a strong possibility that publication bias plays a role in the present results, as publication may be more unlikely for a study that was unable to reject its null hypothesis. In addition, our study focused on VR interventions for victims of crime, a civilian population that may have different characteristics from military samples (e.g., training and deployment), and we did non focus on crimes perpetrated over time (e.g., stalking). Therefore, the treatment population may not be representative of all trauma‐exposed adults. There is a need for more research that includes well‐specified randomization procedures, assessor blinding, larger sample sizes, and intent‐to‐treat analyses.

The findings from this systematic review demonstrate that VR could become a valuable addition to the therapeutic options for traumatized victims of crime. Still, further research is required, preferably in larger groups. This will not only shed more light on the effectiveness of the treatment but also on its place among other interventions, which can enable victims to make more informed decisions about their treatment.

## OPEN PRACTICES STATEMENT

The protocol for this review has been registered in the international prospective register of systematic reviews (PROSPERO registration number CRD42020182543). The list of the excluded studies is on the Supplementary Materials.

## Supporting information

Supplementary Data file 1
Supplementary Data file 2. Studies included – description and results

## References

[jts22810-bib-0001] Alsina‐Jurnet, I. , Gutiérrez‐Maldonado, J. , & Rangel‐Gómez, M. V. (2011). The role of presence in the level of anxiety experienced in clinical virtual environments. Computers in Human Behavior, 27(1), 504–512. 10.1016/j.chb.2010.09.018

[jts22810-bib-0002] Baños, R. M. , Guillen, V. , Quero, S. , García‐Palacios, A. , Alcaniz, M. , & Botella, C. (2011). A virtual reality system for the treatment of stress‐related disorders: A preliminary analysis of efficacy compared to a standard cognitive behavioral program. International Journal of Human‐Computer Studies, 69(9), 602–613. 10.1016/j.ijhcs.2011.06.002

[jts22810-bib-0003] Botella, C. , Fernandez‐Alvarez, J. , Guillen, V. , Garcia‐Palacios, A. , & Banos, R. (2017). Recent progress in virtual reality exposure therapy for phobias: A systematic review. Current Psychiatry Reports, 19(7), 1–13. 10.1007/s11920-017-0788-4 28540594

[jts22810-bib-0004] Botella, C. , García‐Palacios, A. , Guillen, V. , Baños, R. M. , Quero, S. , & Alcaniz, M. (2010). An adaptive display for the treatment of diverse trauma PTSD victims. Cyberpsychology Behavior and Social Networking, 13(1), 67–71. 10.1089/cyber.2009.0353 20528295

[jts22810-bib-0005] Botella, C. , Serrano, B. , Banos, R. M. , & Garcia‐Palacios, A. (2015). Virtual reality exposure‐based therapy for the treatment of post‐traumatic stress disorder: A review of its efficacy, the adequacy of the treatment protocol, and its acceptability. Neuropsychiatric Disease and Treatment, 11, 2533–2545. 10.2147/NDT.S89542 26491332 PMC4599639

[jts22810-bib-0006] Cárdenas‐López, G. , de la Rosa‐Gómez, A. , Durán‐Baca, X. , & Bouchard, S. (2015). Virtual reality PTSD treatment program for civil victims of criminal violence. In P. Cipresso & S. Serino (Eds.), Virtual reality: Technologies, medical applications and challenges (pp. 269–289). Nova Science Publishers.

[jts22810-bib-0007] Cárdenas‐López, G. , de la Rosa, A. , Durón, R. , & Durán, X. (2014, September 2–4). *Virtual reality exposure for trauma and stress‐related disorders for crime victims* [Conference presentation]. The 10^th^ International Conference on Disability, Virtual Reality & Associated Technologies, Gothenburg, Sweden.

[jts22810-bib-0008] Cárdenas‐López, G. , De la Rosa, A. , Lorena, F. , & Ximena, D. (2013, August 26–29). *A controlled trial for PTSD in Mexican victims of criminal violence* [Conference presentation]. 2013 Conference on Virtual Rehabilitation, Philadelphia, PA, United States. 10.1109/ICVR.2013.6662102

[jts22810-bib-0009] Cornet, L. J. M. , & Van Gelder, J. L. (2020). Virtual reality: A use case for criminal justice practice. Psychology, Crime & Law, 26(7), 631–647. 10.1080/1068316x.2019.1708357

[jts22810-bib-0010] Craig, T. , Rus‐Calafell, M. , Ward, T. , Leff, J. P. , Huckvale, M. , Howarth, E. , Emsley, R. , & Garety, P. A. (2018). AVATAR therapy for auditory verbal hallucinations in people with psychosis: A single‐blind, randomised controlled trial. Lancet Psychiatry, 5(1), 31–40. 10.1016/S2215-0366(17)30427-3 29175276 PMC5746597

[jts22810-bib-0011] Difede, J. , Cukor, J. , Jayasinghe, N. , Patt, I. , Jedel, S. , Spielman, L. , Giosan, C. , & Hoffman, H. G. (2007). Virtual reality exposure therapy for the treatment of posttraumatic stress disorder following September 11, 2001. Journal of Clinical Psychiatry, 68(11), 1639–1647. 18052556

[jts22810-bib-0012] Difede, J. , Cukor, J. , Wyka, K. , Olden, M. , Hoffman, H. , Lee, F. S. , & Altemus, M. (2014). D‐cycloserine augmentation of exposure therapy for post‐traumatic stress disorder: A pilot randomized clinical trial. Neuropsychopharmacology, 39(5), 1052–1058. 10.1038/npp.2013.317 24217129 PMC3957110

[jts22810-bib-0013] Eichenberg, C. (2010). Application of “virtual realities” in psychotherapy: Possibilities, limitations and effectiveness. IntechOpen. 10.5772/12914

[jts22810-bib-0014] Ferrer‐García, M. , & Gutiérrez‐Maldonado, J. (2012). The use of virtual reality in the study, assessment, and treatment of body image in eating disorders and nonclinical samples: Aa review of the literature. Body Image, 9(1), 1–11. 10.1016/j.bodyim.2011.10.001 22119329

[jts22810-bib-0015] Fodor, L. A. , Coteț, C. D. , Cuijpers, P. , Szamoskozi, Ș. , David, D. , & Cristea, I. A. (2018). The effectiveness of virtual reality‐based interventions for symptoms of anxiety and depression: A meta‐analysis. Scientific Reports, 8(1), 1–13. 10.1038/s41598-018-28113-6 29985400 PMC6037699

[jts22810-bib-0016] Freeman, D. , Antley, A. , Ehlers, A. , Dunn, G. , Thompson, C. , Vorontsova, N. , Garety, P. , Kuipers, E. , Glucksman, E. , & Slater, M. (2014). The use of immersive virtual reality (VR) to predict the occurrence 6 months later of paranoid thinking and posttraumatic stress symptoms assessed by self‐report and interviewer methods: A study of individuals who have been physically assaulted. Psychological Assessment, 26(3), 841–847. 10.1037/a0036240 24708073 PMC4151801

[jts22810-bib-0017] Fromberger, P. , Jordan, K. , & Muller, J. L. (2018). Virtual reality applications for diagnosis, risk assessment, and therapy of child abusers. Behavioral Sciences & the Law, 36(2), 235–244. 10.1002/bsl.2332 29520819

[jts22810-bib-0018] Fromberger, P. , Meyer, S. , Jordan, K. , & Muller, J. L. (2018). Behavioral monitoring of sexual offenders against children in virtual risk situations: A feasibility study. Frontiers in Psychology, 9(224), 1–17. 10.3389/fpsyg.2018.00224 29559934 PMC5845629

[jts22810-bib-0019] Goncalves, R. , Pedrozo, A. L. , Coutinho, E. S. , Figueira, I. , & Ventura, P. (2012). Efficacy of virtual reality exposure therapy in the treatment of PTSD: A systematic review. PLoS One, 7(12), 1–7. 10.1371/journal.pone.0048469 PMC3531396 23300515

[jts22810-bib-0020] Higgins, J. P. , & Green, S. (2011). *Cochrane Handbook for systematic reviews of interventions* (Version 5.1.0). The Cochrane Collaboration.

[jts22810-bib-0021] Jouriles, E. N. , Simpson Rowe, L. , McDonald, R. , & Kleinsasser, A. L. (2014). Women's expression of anger in response to unwanted sexual advances: Associations with sexual victimization. Psychology of Violence, 4(2), 170–183. 10.1037/a0033191

[jts22810-bib-0022] Kellmeyer, P. , Biller‐Andorno, N. , & Meynen, G. (2019). Ethical tensions of virtual reality treatment in vulnerable patients. Nature Medicine, 25(8), 1185–1188. 10.1038/s41591-019-0543-y 31359003

[jts22810-bib-0023] Keswani, B. , Mohapatra, A. G. , Mishra, T. Ch. , Keswani, P. , Mohapatra, P. Ch. G. , Akhtar, M. M. , & Vijay, P. (2020). World of virtual reality (VR) in health care. In G. Deepak , A. Hassanien , & A. Khanna (Eds), Advanced computational intelligence techniques for virtual reality in health care. Springer. 10.1007/978-3-030-35252-3_1

[jts22810-bib-0024] Kip, H. , Bouman, Y. H. A. , Kelders, S. M. , & van Gemert‐Pijnen, L. (2018). eHealth in treatment of offenders in forensic mental health: A review of the current state. Frontiers in Psychiatry, 9(42), 1–19. 10.3389/fpsyt.2018.00042 29515468 PMC5826338

[jts22810-bib-0025] Kip, H. , Kelders, S. M. , Bouman, Y. H. A. , & van Gemert‐Pijnen, L. (2019). The importance of systematically reporting and reflecting on eHealth development: Participatory development process of a virtual reality application for forensic mental health care. Journal of Medical Internet Research, 21(8), 1–16. 10.2196/12972 PMC6718085 31429415

[jts22810-bib-0026] Kip, H. , Kelders, S. M. , Weerink, K. , Kuiper, A. , Bruninghoff, I. , Bouman, Y. H. A. , Dijkslag, D. , & van Gemert‐Pijnen, L. (2019). Identifying the added value of virtual reality for treatment in forensic mental health: A scenario‐based, qualitative approach. Frontiers in Psychology, 10(406), 1–15. 10.3389/fpsyg.2019.00406 30873093 PMC6400887

[jts22810-bib-0027] Klein Tuente, S. , Bogaerts, S. , Bulten, E. , Keulen‐de Vos, M. , Vos, M. , Bokern, H. , van IJzendoorn, S. , Geraets, C. N. W. , & Veling, W. (2020). Virtual reality aggression prevention therapy (VRAPT) versus waiting list control for forensic psychiatric inpatients: A multicenter randomized controlled trial. Journal of Clinical Medicine, 9(7), 1–18. 10.3390/jcm9072258 PMC7409015 32708637

[jts22810-bib-0028] Liberati, A. , Altman, D. G. , Tetzlaff, J. , Mulrow, C. , Gøtzsche, P. C. , Ioannidis, J. P. A. , Clarke, M. , Devereaux, P. J. , Kleijnen, J. , & Moher, D. (2009). The PRISMA statement for reporting systematic reviews and meta‐analyses of studies that evaluate health care interventions: Explanation and elaboration. PLoS Medicine, 6(7), 1–28. 10.1371/journal.pmed.1000100 PMC2707010 19621070

[jts22810-bib-0029] Loucks, L. , Yasinski, C. , Norrholm, S. D. , Maples‐Keller, J. , Post, L. , Zwiebach, L. , Fiorillo, D. , Goodlin, M. , Jovanovic, T. , Rizzo, A. A. , & Rothbaum, B. O. (2019). You can do that?!: Feasibility of virtual reality exposure therapy in the treatment of PTSD due to military sexual trauma. Journal of Anxiety Disorders, 61, 55–63. 10.1016/j.janxdis.2018.06.004 30005843

[jts22810-bib-0030] Loranger, C. , & Bouchard, S. (2017). Validating a virtual environment for sexual assault victims. Journal of Traumatic Stress, 30(2), 157–165. 10.1002/jts.22170 28422323

[jts22810-bib-0031] Motraghi, T. E. , Seim, R. W. , Meyer, E. C. , & Morissette, S. B. (2014). Virtual reality exposure therapy for the treatment of posttraumatic stress disorder: A methodological review using CONSORT guidelines. Journal of Clinical Psychology, 70(3), 197–208. 10.1002/jclp.22051 24108479

[jts22810-bib-0032] Nee, C. , Gelder, J. , Otte, M. , Vernham, Z. , & Meenaghan, A. (2019). Learning on the job: Studying expertise in residential burglars using virtual environments. Criminology; An Interdisciplinary Journal, 57, 481–511. 10.1111/1745-9125.12210

[jts22810-bib-0033] Nee, C. , White, M. , Woolford, K. , Pascu, T. , Barker, L. , & Wainwright, L. (2014). New methods for examining expertise in burglars in natural and simulated environments: Preliminary findings. Psychology, Crime & Law, 21(5), 507–513. 10.1080/1068316x.2014.989849

[jts22810-bib-0034] Parsons, T. D. , Gaggioli, A. , & Riva, G. (2017). Virtual reality for research in social neuroscience. Brain Sciences, 7(4), 1–21. 10.3390/brainsci7040042 PMC5406699 28420150

[jts22810-bib-0035] Peskin, M. , Wyka, K. , Cukor, J. , Olden, M. , Altemus, M. , Lee, F. S. , & Difede, J. (2019). The relationship between posttraumatic and depressive symptoms during virtual reality exposure therapy with a cognitive enhancer. Journal of Anxiety Disorder, 61, 82–88. 10.1016/j.janxdis.2018.03.001 29580634

[jts22810-bib-0036] Renaud, P. , Rouleau, J. L. , Proulx, J. , Trottier, D. , Goyette, M. , Bradford, J. P. , Fedoroff, P. , Dufresne, M. ‐H. , Dassylva, B. , Côté, G. , & Bouchard, S. (2010). Virtual characters designed for forensic assessment and rehabilitation of sex offenders: Standardized and made‐to‐measure. Journal of Virtual Reality and Broadcasting, 7(5), 10.20385/1860-2037/7.2010.5

[jts22810-bib-0037] Schuemie, M. J. , van der Straaten, P. , Krijn, M. , & van der Mast, C. A. (2001). Research on presence in virtual reality: A survey. Cyberpsychology & Behavior, 4(2), 183–201. 10.1089/109493101300117884 11710246

[jts22810-bib-0038] Sterne, J. A. , Hernan, M. A. , Reeves, B. C. , Savović, J. , Berkman, N. D. , Viswanathan, M. , Henry, D. , Altman, D. G. , Ansari, M. T. , Boutron, I. , Carpeter, J. R. , Chan, A. , Churchill, R. , Deeks, J. J. , Hróbjartsson, A. , Kirkham, J. , Jüni, P. , Loke, Y. L. , Pigott, T. D. , … Higgins, J. P. T. (2016). ROBINS‐I: A tool for assessing risk of bias in non‐randomised studies of interventions BMJ, 355, i4919. 10.1136/bmj.i4919 27733354 PMC5062054

[jts22810-bib-0039] van Rijn, B. , Cooper, M. , Jackson, A. , & Wild, C. (2015). Avatar‐based therapy within prison settings: Pilot evaluation. British Journal of Guidance & Counselling, 45(3), 268–283. 10.1080/03069885.2015.1068273

[jts22810-bib-0040] Veling, W. , Moritz, S. , & van der Gaag, M. (2014). Brave new worlds—review and update on virtual reality assessment and treatment in psychosis. Schizophrenia Bulletin, 40(6), 1194–1197. 10.1093/schbul/sbu125 25193975 PMC4193729

