# Supplementary material for: Virtual reality interventions for victims of crime: A systematic review
Source: J Trauma Stress. 2022 Feb 28;35(3):804–12. doi: 10.1002/jts.22810 (PMC9306974; doi:10.1002/jts.22810)
Supplement: Supplementary file 1 — Supplementary Data file 1 Supplementary Data file 2. Studies included – description and results [file JTS-35-804-s001.docx]

# Supplementary Data file 1

| Excluded studies **Study** | **Reason for exclusion** |
| --- | --- |

| 1. Seinfeld S, Arroyo-Palacios J, Iruretagoyena G, Hortensius R, Zapata LE, Borland D, de Gelder B, Slater M, Sanchez-Vives MV. (2018). Offenders become the victim in virtual reality: impact of changing perspective in domestic violence. Sci Rep.;8(1):2692. | Wrong population |
| --- | --- |
| 1. Valmaggia LR, Day FL, Kroll J, Laing J, Byrne M, Fusar-Poli P, McGuire P. (2015). Bullying victimization and paranoid ideation in people at ultra high risk for psychosis. Schizophr Res. 168(1-2):68-73. | Duplicated same data |
| 1. de Borst AW, Sanchez-Vives MV, Slater M, de Gelder B. (2020). First person virtual embodiment modulates cortical network that encodes the bodily self and its surrounding space during the experience of domestic violence. eNeuro. ENEURO.0263-19.2019. | Wrong population |
| 1. Neyret S, Navarro X, Beacco A, Oliva R, Bourdin P, Valenzuela J, Barberia I, Slater M. An Embodied Perspective as a Victim of Sexual Harassment in Virtual Reality Reduces Action Conformity in a Later Milgram Obedience Scenario. Sci Rep. 2020 Apr 10;10(1):6207. | Wrong population |
| 1. Rowe LS, Jouriles EN, McDonald R. (2015). Reducing sexual victimization among adolescent girls: a randomized controlled pilot trial of my voice, my choice. Behav Ther. 2015 May;46(3):315-27. | Wrong population |
| 1. Corno, Giulia; Bouchard, Stéphane. An innovative positive psychology VR application for victims of sexual violence: A qualitative study. *In:*Virtual reality: Technologies, medical applications and challenges. Cipresso, Pietro (Ed); Serino, Silvia (Ed); Publisher: Nova Science Publishers; 2015, pp. 229-267. [Chapter] | Wrong design |
| 1. Toet, Alexander; van Schaik, Martin G. (2012); Effects of signals of disorder on fear of crime in real and virtual environments. Journal of Environmental Psychology, Vol 32(3), Sep, 2012 pp. 260-276. | Wrong population |
| 1. Cornet, L. J., & Van Gelder, J. L. (2020). Virtual reality: a use case for criminal justice practice. Psychology, Crime & Law, 26(7), 631-647. | Wrong design |
| 1. Banks, James. (2013). Edging your bets: Advantage play, gambling, crime and victimisation. **Crime**, Media, Culture, Vol 9(2), Aug, 2013 pp. 171-187. | Wrong design |
| 1. Dremliuga, R. I., Mamychev, A. Y., Dremliuga, O. A., & Matyuk, Y. S. (2019). Crimes in Virtual Reality. Dilemas Contemporáneos: Educación, Política y Valores, 7(1). | Wrong design |
| 1. Ingram, K. M., Espelage, D. L., Merrin, G. J., Valido, A., Heinhorst, J., & Joyce, M. (2019). Evaluation of a virtual reality enhanced bullying prevention curriculum pilot trial. Journal of adolescence, 71, 72-83. | Wrong population |
| 1. Reger, G. M., Koenen-Woods, P., Zetocha, K., Smolenski, D. J., Holloway, K. M., Rothbaum, B. O., ... & Gahm, G. A. (2016). Randomized controlled trial of prolonged exposure using imaginal exposure vs. virtual reality exposure in active duty soldiers with deployment-related posttraumatic stress disorder (PTSD). Journal of consulting and clinical psychology, 84(11), 946. | Wrong population |
| 1. Jouriles, E. N., McDonald, R., Kullowatz, A., Rosenfield, D., Gomez, G. S., & Cuevas, A. (2009). Can virtual reality increase the realism of role plays used to teach college women sexual coercion and rape-resistance skills?. Behavior therapy, 40(4), 337-345. | Wrong population |
| 1. Van Den Broeck, E., Poels, K., Vandebosch, H., Van Royen, K. Online perspective-taking as an intervention tool against cyberbullying. (2014) Annual Review of CyberTherapy and Telemedicine, 12, pp. 113-117. | Wrong population |
| 1. Kyriakou, M., & Chrysanthou, Y. (2018, November). How responsiveness, group membership and gender affect the feeling of presence in immersive virtual environments populated with virtual crowds. In Proceedings of the 11th annual international conference on motion, interaction, and games (pp. 1-9). | Wrong design |
| 1. Ólafsdóttir, H.M., Ómarsdóttir, E., Saeland, H., Vilhjálmsson, H.H. Work in progress report: Virtual courtroom to prepare victims of sex crimes for court proceedings chiesto su researchgate. Proceedings of the 19th ACM International Conference on Intelligent Virtual Agents, pp. 170-172. | Wrong design |
| 1. Giulia, C., Stéphane, B. An Innovative Positive Psychology Vr Application For Victims Of Sexual Violence: A Qualitative Study. Virtual Reality: Technologies, Medical Applications and Challenges, pp. 229-267. | Wrong design |
| 1. Freeman D, Thompson C, Vorontsova N, Dunn G, Carter LA, Garety P, Kuipers E, Slater M, Antley A, Glucksman E, Ehlers A. (2013) Paranoia and post-traumatic stress disorder in the months after a physical assault: a longitudinal study examining shared and differential predictors. Psychol Med.43(12):2673-84 | Duplicated data |
| 1. Sapouna, M., Wolke, D., Vannini, N., Watson, S., Woods, S., Schneider, W., ... & Aylett, R. (2010). Virtual learning intervention to reduce bullying victimization in primary school: a controlled trial. Journal of Child Psychology and Psychiatry, 51(1), 104-112. | Wrong population |
| 1. De la Rosa A, Cárdernas-López G. Posttraumatic stress disorder:efficacy of a treatment program using virtual reality for victims of criminal violence in Mexican population. Anu   Psicología 2012;42:377–91. | Duplicated data |
| 1. Jouriles EN, Simpson Rowe L, McDonald R, Platt CG, Gomez GS. (2011). Assessing women's responses to sexual threat: validity of a virtual role-play procedure. Assessing women's responses to sexual threat: validity of a virtual role-play procedure. Behav Ther. 2011 Sep;42(3):475-84. | Duplicated data |
| 1. Cárdenas-López, G., de la Rosa, A., Durón, R., & Durán, X. (2016). Virtual reality exposure for trauma and stress-related disorders for city violence crime victims. International Journal of Child Health and Human Development, 9(3), 315-322. | Duplicated data |
| 1. Cárdenas, G., & De La Rosa, A. (2012). Using virtual reality for PTSD treatment in criminal violence victims. Journal of CyberTherapy & Rehabilitation, 5(1), 65-67 | Author did not provide data |
| 1. McDonnell, J., Stahl, D., Day, F., McGuire, P., & Valmaggia, L. R. (2018). Interpersonal sensitivity in those at clinical high risk for psychosis mediates the association between childhood bullying victimisation and paranoid ideation: a virtual reality study. Schizophrenia research, 192, 89-95. | Wrong population |
| 1. R. M. Baños, V. Guillen, S. Quero, A. García-Palacios, M. Alcaniz, and C. Botella. 2011. A virtual reality system for the treatment of stress-related disorders: A preliminary analysis of efficacy compared to a standard cognitive behavioral program. Int. J. Hum.-Comput. Stud. 69, 9 (August, 2011), 602–613. DOI:https://doi.org/10.1016/j.ijhcs.2011.06.002 | Wrong population |

# Supplementary Data file 2

***Studies included – description and results***

| Study | Focus | Population | Intervention groups | N | Follow-up | Drop out | Relevant outcomes (measures) | Results |
| --- | --- | --- | --- | --- | --- | --- | --- | --- |
| Botella et al, 2010 | Study the efficacy of a VR in the treatment of trauma PTSD victims | Trauma PTSD victims | CBT  CBT + VR adaptive display (EMMA’s World) | 10 (5 CBT, 5 CBT + EMMA’s world) | 9 weeks** | Not reported | Treatment outcome (CAPS; DTS; PTCI) | No significant differences in CAPS, DTS and PTCI scores between the two groups |
| Cardenas-Lopez et al., 2013 | Study the efficacy of  VRET for PTSD for criminal violence | Victims and witnesses  of crime with PTSD | VRET  IET | 20 (10 VRET, 10 IET) | 12 weeks | 10 VRET  10 IET | Treatment outcome (CAPS-1; PSSSR)  Secondary outcome (STAI; BDI)  Patient satisfaction (Treatment satisfaction questionnaire) | No significant differences in primary and secondary outcome, except for the CAPS-1 avoidance showing a higher therapeutic profiting in VRET. |
| Cardenas-Lopez et al., 2014 | Study the efficacy of VRET for PTSD and ASD for crime violence | Victims and witnesses of crime with PTSD or ASD | VRET | 9 (6 with PTSD, 3 with ASD) | 12 weeks | Not reported | Treatment outcome (CAPS; PSSSR)  Secondary outcome (STAI; BDI)  Patient satisfaction (Treatment satisfaction questionnaire) | All patients showed an improvement (>30 percent) in their PTSD, anxiety depressive symptoms and were satisfied |
| Cardenas-Lopez et al., 2015*** | Study the efficacy of VRET for PTSD for crime violence | Victims and witnesses of crime | VRET | 5 | 12 weeks | Not reported | Treatment outcome (CAPS-1; PSSSR)  Secondary outcome (BAI; BDI) | All patients obtained clinical change for CAPS-1, CAPS-1 avoidance, PSSSR,  and BDI |
| Difede et al., 2007 | Study the efficacy of VR exposure therapy for PTSD | Civilians and disaster workers exposed at the World Trade Center attacks of September 2001 | VR  Waitlist control | 18 (10 VR, 8 waitlist) | 6 months | 3 VR | Treatment outcome (CAPS)  Secondary outcome (BDI; GSI) | Significant reduction in CAPS scores in patients in the VR group |
| Freeman et al., 2014**** | To assess the occurrence of paranoia and PTSD symptoms in a neutral VR social environment as predictors of later psychiatric symptoms | Victims of assault | Neutral VR social  environment (a train ride) | 106 | 6 months | Not reported | Treatment outcome (PANSS; GPTS; VAS Paranoia; PSSI; PDS; SSPS; VR PTSD; Visual analogue rating scales) | Responses to VR predicted the severity of paranoia and PTSD symptoms as assessed by standard measures 6 months later |
| Jouriles et al., 2014 | To evaluate whether VR can enhance the realism of role plays designed to help college women resist sexual attacks | Female undergraduate students with and without history of sexual victimization | VR role play with male actors making unwanted sexual advances toward the participant | 65 | --- | Not reported | Treatment outcome (Sexual victimization; Observed anger; Anger words; Assertive resistance; Immersion; Actor aggression) | Women with a history of sexual victimization were less angry and used fewer anger words in response to unwanted sexual advances |
| Loranger et al., 2017 | To assess the relevance and safety of a VE ì progressively exposing to a sexual assault scenario | Women (victims  and nonvictims of sexual assault) | VE dealing with  a sexual assault scenario (EXP)  VE without a sexual assault scenario (CONT) | 30 (15 CONT-EXP, 15 EXP-CONT) | --- | Not reported | Treatment outcome (ADIS-IV; BDI; THS; STAI; PANAS; SUDS) | Participants experienced significantly more anxiety and negative affect in the experimental scenario than in the control condition |
| Peskin et al., 2019***** | To explore the temporal relationship between posttraumatic and depressive symptoms in a randomized trial DCS versus placebo augmented VRE therapy for chronic PTSD | Subjects with chronic World Trade Center-related PTSD following terrorist attacks | VR + DCS  Virtual reality + placebo (VR – placebo) | 25 (13 VR-DCS, 12 VR – placebo) | 12 weeks | 3 VR placebo | Treatment outcome (PCL;  BDI-II) | VR primarily decreases posttraumatic symptoms, which in turn leads to decreased depressive symptoms, and DCS may strengthen these effects |

Note: DCS: D-Cycloserine; VR: Virtual Reality; PTSD: Post Traumatic Stress Disorder; CBT: Cognitive Behavior therapy; CAPS: Clinician-Administered PTSD Scale; DTS: Davidson Trauma Scale; PTCI: Posttraumatic Cognitions Inventory; VRET: Virtual reality prolonged exposure therapy; IET: imaginal prolonged exposure therapy; PSSSR: Posttraumatic Stress Symptom Scale, Self-Report; STAI: State-Trait Anxiety Inventory; BDI: Beck Depression Inventory; ASD: Acute Stress Disorder; BAI: Beck Anxiety Inventory; GSI: Global Severity Inventory; PANSS: Positive and Negative Symptom Scale; GPTS: Green et al. Paranoid Thoughts Scale; VAS: Visual Analogue Scale; PSSI: Interviewer version of the PTSD Symptom Scale; PDS: Posttraumatic Diagnostic Scale; SSPS: State Social Paranoia Scale; VR PTSD: assessment of PTSD symptoms during the virtual reality experience; VE: virtual environment; EXP: experimental; CONT: control; ADIS-IV: Anxiety Disorders Interview Schedule for DSM-IV; THS: Trauma History Screen; PANAS: Positive and Negative Affect Schedules; SUDS: Subjective Units of Discomfort Scores; PCL: PTSD Checklist

* For PTSD patients, the traumatic experiences were abuse, crime assault and car accident; ** participants who needed more sessions could receive treatment for 3 additional weeks; *** book chapter; ****integrated with Freeman et al, 2013; *****integrated with Difede et al, 2014
